# Sensory integration in a department of child and adolescent psychiatry

**DOI:** 10.1192/j.eurpsy.2023.848

**Published:** 2023-07-19

**Authors:** M. L. Maria Del Carmen, B. B. Nadia, C. I. Irene

**Affiliations:** 1 Psiquiatría, Hospital General Universitario Gregorio Marañón; 2 Psiquiatría, Hospital Universitario de Torrejón, Madrid, Spain

## Abstract

**Introduction:**

The area of Sensory Integration has its origin in the 1960s, developed by the neuroscientist and occupational therapist Jean Ayres. Although the first studies focused on the relationship between learning problems and atypical sensory processing, today there are new applications in clinical practice.

Sensory integration is defined as the neurological process responsible for organizing the sensations that one receives from one’s own body and from the environment, in order to respond and function adequately in relation to environmental demands.

**Objectives:**

This work has several objectives. On the one hand, review the concept of sensory integration, the definition and theoretical basis as well as the scientific evidence of this theory. On the other hand, review the use of sensory integration in psychiatric practice from the 1960s to the present day. Also, explain the experience of a child and adolescent psychiatry unit with the use of sensory integration as part of the treatment. Finally, new challenges, approaches and needs of psychiatry services will be considered for the implementation or improvement of this new work tool in a multidisciplinary team.

**Methods:**

A bibliographic search has been carried out in the main sources of medical information such as pubmed, uptodate as well as in national and international journals. Likewise, the knowledge and clinical experience of the team has been reviewed.

**Results:**

In our clinical experience, the child and adolescent psychiatry device for intensive outpatient treatment where patients between 12 and 17 years of age with severe mental disorders attend, initially passed the sensory profile by occupational therapy to patients who presented behavioral or emotional symptoms. not consistent with the psychopathological examination. In view of the results and magnificent progress, this intervention began to be carried out systematically to the boys who joined the device.We present the case of a 15-year-old patient who attended the device due to emotional dysregulation and suicidal risk. During evolution, possible difficulties were seen in sensory integration that made it difficult for the patient to improve with psychiatric or psychological therapy alone. The patient was evaluated and treated by the team’s occupational therapist, specifically trained in sensory integration. It was evaluated with the sensory profile, with the results having a sensory sensitivity profile and auditory and tactile avoidance. The specific measures that were carried out were: sensory diet and environmental modifications.

**Conclusions:**

Sensory integration is a therapy with sufficient clinical evidence to implement it in child and adolescent psychiatry services. Therapy should be performed by suitably trained and validated occupational therapists. This therapy must be included in a multidisciplinary approach to the patient and specific modifications that can be developed at home and at school are provided.

**Disclosure of Interest:**

None Declared

